# Moderate Intra-Abdominal Hypertension Leads to Anaerobic Metabolism in the Rectus Abdominis Muscle Tissue of Critically Ill Patients: A Prospective Observational Study

**DOI:** 10.1155/2014/857492

**Published:** 2014-03-13

**Authors:** Liivi Maddison, Juri Karjagin, Jyrki Tenhunen, Ülle Kirsimägi, Joel Starkopf

**Affiliations:** ^1^University of Tartu, Tartu University Hospital, Clinic of Anaesthesiology and Intensive Care, Puusepa 8, 51014 Tartu, Estonia; ^2^Critical Care Medicine Research Group, Department of Intensive Care Medicine, Tampere University Hospital, Teiskontie 35, PL 2000, 33521 Tampere, Finland; ^3^Department of Surgical Sciences/Anaesthesiology and Intensive Care Medicine, University of Uppsala, Akademiska sjukhuset Building 70, 1 tr, 751 85 Uppsala, Sweden; ^4^University of Tartu, Tartu University Hospital, Clinic of Surgery, Puusepa 8, 51014 Tartu, Estonia

## Abstract

*Purpose*. We hypothesize that intra-abdominal hypertension (IAH) is associated with the presence of anaerobic metabolism in the abdominal rectus muscle (RAM) tissue of critically ill patients. *Methods*. We included 10 adult, critically ill patients with intra-abdominal pressure (IAP) above 12 mmHg. Microdialysis catheters (CMA 60) were inserted into the RAM tissue. The samples were collected up to 72 hours after enrollment. *Results*. The patients' median (IQR) APACHE II at inclusion was 29 (21–37); 7 patients were in shock. IAP was 14.5 (12.5–17.8) mmHg at baseline and decreased significantly over time, concomitantly with arterial lactate and vasopressors requirements. The tissue lactate-to-pyruvate (L/P) ratio was 49 (36–54) at the beginning of the study and decreased significantly throughout the study. Additionally, the tissue lactate, lactate-to-glucose (L/G) ratio, and glutamate concentrations changed significantly during the study. The correlation analysis showed that lower levels of pyruvate and glycerol were associated with higher MAP and abdominal perfusion pressures (APP) and that higher levels of glutamate were correlated to elevated IAP. *Conclusions*. Moderate IAH leads to RAM tissue anaerobic metabolism suggestive for hypoperfusion in critically ill patients. Correlation analysis supports the concept of using APP as the primary endpoint of resuscitation in addition to MAP and IAP.

## 1. Introduction

Intra-abdominal hypertension (IAH), defined as a sustained intra-abdominal pressure (IAP) above 12 mmHg, directly affects splanchnic perfusion and is one of the triggers for multiple organ failure [1]. IAH, which affects approximately one-third of intensive care patients, is an independent predictor for mortality [2–4]. In up to 8% of IAH cases, IAH manifests as life-threatening abdominal compartment syndrome (ACS); its treatment has been increasingly addressed in the past [5, 6]. Consequently, the mortality of ACS has decreased approximately two-fold in the last decade [5]. However, there is still a “gray zone” in IAP, ranging from 12 mmHg to 18 mmHg, whereby the clinical consequences of IAH are not clearly evident and treatment recommendations are inconsistent. Thus, there is no clear trigger at which level of IAP active treatment should commence.

We previously demonstrated that short-term increase of IAP to 12 mmHg results in increased lactate-pyruvate (L/P) ratio in the abdominal rectus muscle (RAM) of laparoscopic surgical patients, which is detected using microdialysis (MD) [7]. This finding suggests that the deterioration of tissue metabolism in the abdominal area may occur well before the clinical signs of organ dysfunctions related to IAH are evident. Recent animal experiments further support this notion and suggest that microdialysis of RAM may serve as an easily accessible site for the early detection of subclinical organ dysfunction [8]. This finding, however, has not been investigated in critically ill patients. Therefore, the present study was undertaken to test the hypothesis that elevated IAP and decreased abdominal perfusion pressure (APP) are associated with tissue hypoperfusion and the prevalence of anaerobic metabolism in RAM tissue in critically ill patients.

## 2. Materials and Methods

The study was approved by the University of Tartu Ethics Review Committee on Human Research (Protocol Number 170/T-11 28.04.2008). The study was performed according to the Declaration of Helsinki.

### 2.1. Study Design

This study was a prospective, single-center, and observational study.

### 2.2. Patients

Adult, mechanically ventilated patients who were admitted to the Department of General Intensive Care (ICU) at Tartu University Hospital were screened for study inclusion during the first 3 days of their treatment. The patients were eligible for the study if they were ≥18 years of age, if the IAP measurement was possible (i.e., the urinary bladder catheter had been positioned), and if they had at least one of the following risk factors for the development and presence of IAH at the time of ICU admission:

- acute pancreatitis;
- liver failure with cirrhosis and ascites;
- gastrointestinal hemorrhage;
- use of vasopressors and inotropic agents;
- PaO_2_/FiO_2_ ≤ 300 mmHg.

These criteria were previously identified as risk factors for IAH in adult, mechanically ventilated, ICU patients [4]. The current study was performed in the same department; therefore, the case-mix was similar.

After the patients provided informed consent, 10 patients (1 female, 9 males) with a median age of 65 (range, 19–89) years were studied. The reasons for ICU admission were postresuscitative state (*n* = 2 patients), gastrointestinal bleeding (*n* = 2 patients), ruptured abdominal aortic aneurysm, acute pancreatitis, multiple trauma, methanol intoxication, cardiac failure, and tetanus.

In all of the enrolled patients, the IAP measurements were performed at approximately 6-hour intervals. IAP was measured via the patient's urinary bladder catheter at end-expiration with the patients in the supine position using commercially available Foley catheter kits from Unomedical (Birkerød, Denmark). The system is based on a revised closed-system, repeated measurement technique [9]. An instillation volume of 25 mL was used and the mid-axillary line was noted as a zero level for IAP readings [10]. The patients were sedated if necessary (in addition to baseline sedation) during the IAP measurements to avoid excessive pressure artifacts. If the patient had IAP ≥ 12 mmHg for at least 12 hours, she or he was included in the study.

Exclusion criteria were as follows: patient's or next-of-kin's refusal to participate, abdominal surgery, and BMI ≥ 32 kg/m^2^.

### 2.3. Microdialysis

At the beginning of a study, an MD catheter (CMA 60, Solna, Sweden) was inserted into the RAM. Catheter placement was confirmed using ultrasound (Sonosite MicroMaxx, Bothell, WA, USA). The microdialysate perfusion rate was set at 0.3 *μ*L/min. The sample specimens were collected at 26 time points: hourly for the first eight hours, every two hours for the next eight hours, and then every four hours until the end of the study. The duration of the study was 72 hours. The sample specimens were stored in a freezer at −80°C at Tartu University Hospital in Estonia and were sent in a single shipment to Tampere University Hospital in Finland for additional analyses. Glucose, lactate, pyruvate, glycerol, and glutamate concentrations of the microdialysates were measured using a CMA 600 analyzer (Solna, Sweden). The lactate-to-pyruvate ratio (L/P ratio) and lactate-to-glucose ratio (L/G ratio) were calculated.

### 2.4. Statistical Analysis

Statistical analyses were performed using GraphPad Prism 5.02 (GraphPad Software, Inc., San Diego, CA, USA). Normality of distribution was tested using the Kolmogorov-Smirnov test. For the RAM tissue metabolite concentrations (not normally distributed data), Friedman's test was used to test the change over the observation period. The patients' clinical characteristics were averaged for corporate 6 time points (baseline and from 3 to 6 hours, 7 to 12 hours, 12 to 24 hours, 24 to 48 hours, and 48 to 72 hours) and analyzed using a repeated-measurements analysis of variance (normally distributed data) or Friedman's test. For the post hoc analysis, Dunn's test was used.

For correlation analysis, consecutive single measurements were used. Analysis was performed using the STATISTICA 10 (Software System Statsoft, Inc., Tulsa, OK, USA) software; correlations within the subjects (using the method by Bland and Altman) were employed because of the presence of multiple measurements from one patient [11]. To remove the variation caused by the subjects, we performed an analysis of covariance to evaluate the relationship between the microdialysate contents and clinical variables; all of the patients were simultaneously treated as a categorical factor. Because the majority of the data were not normally distributed, they were presented as medians with interquartile ranges (IQR). The differences were considered to be significant at *P* ≤ 0.05.

## 3. Results

Seven of 10 patients were in shock at the beginning of the study: 2 patients were in septic shock, 2 patients were in cardiogenic shock, 2 patients were in hemorrhagic shock, and 1 patient was in distributive (methanol poisoning) shock. One patient died 16 hours after being included in the study; the remaining patients were discharged alive from the ICU. The median length of stay in the ICU was 11 (8–18) days. The patients' median APACHE II score at the time of inclusion in the study was 29 (21–37).

The patients' clinical characteristics are presented in Table 1.

**Figure 1:**
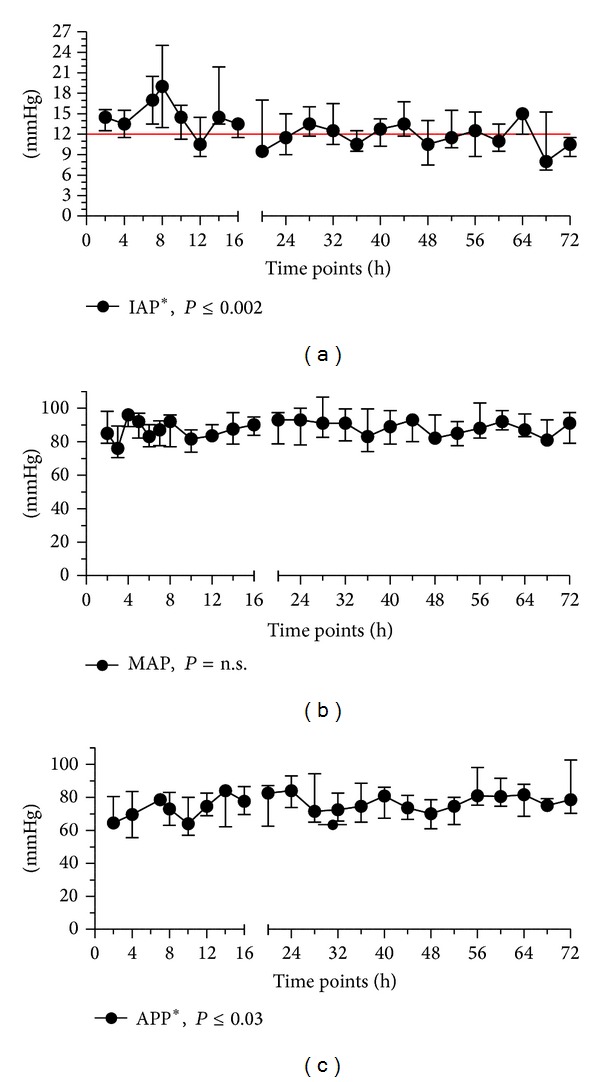
Median changes in the intra-abdominal pressure (IAP), mean arterial pressure (MAP), and abdominal perfusion pressure (APP) during the study period. *IAP decreased significantly and APP increased throughout the observation period (*P* ≤ 0.05). Bars indicate interquartile ranges.

IAP was moderately elevated at baseline (14.5 (12.5–17.8) mmHg) and decreased significantly over the observation period (*P* = 0.002) (Figure 1). The mean arterial pressure (MAP) remained stable throughout the study (Figure 1), whereas arterial lactate and vasopressor requirements decreased significantly, indicating the appropriate treatment of shock in our patients. APP (calculated as the difference between the MAP and IAP) increased significantly during the study period (Figure 1); however, the median value was never below 60 mmHg, which is considered to be a minimally sufficient level for visceral perfusion [12].

### 3.1. Glucose, Pyruvate, Lactate, and L/P and L/G Ratios

**Figure 2:**
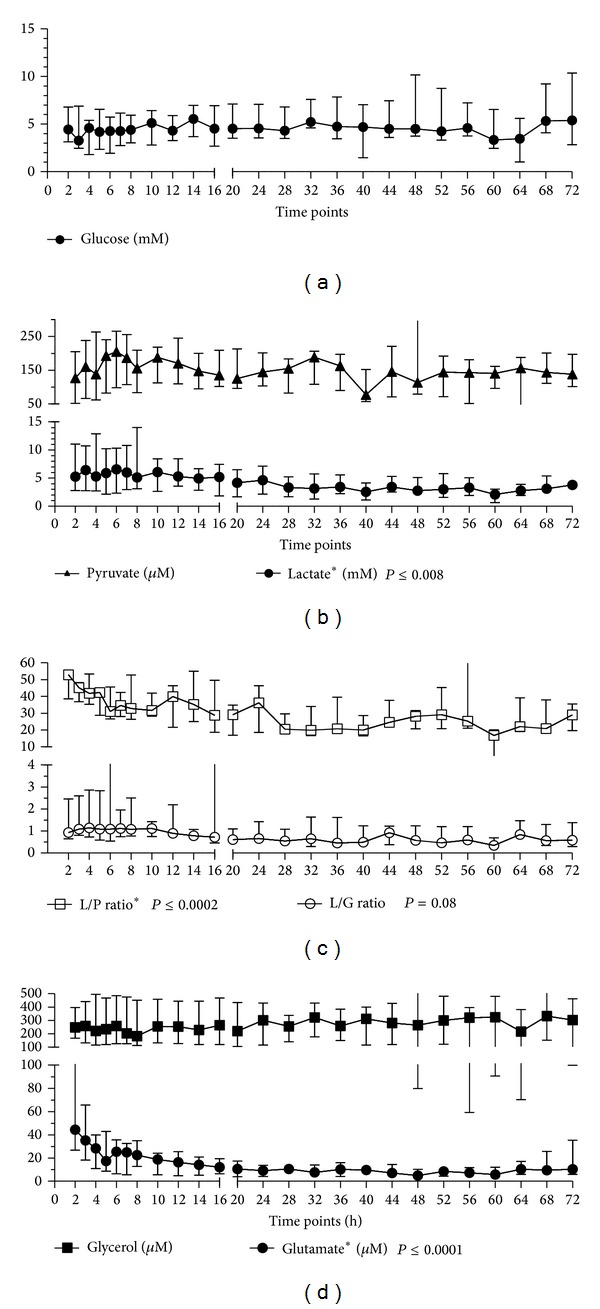
Dynamic of tissue metabolite in abdominal rectus muscle (RAM). L/P ratio, lactate-to-pyruvate ratio; L/G ratio, lactate-to-glucose ratio. *Significant changes throughout the observation period.

The baseline value of tissue glucose was 4.1 (3.2–6.5) mM, pyruvate was 129 (57–189) *μ*M, and lactate was 4.7 (2.7–10) mM. The baseline L/P ratio was 49 (36–54) and the L/G ratio was 0.93 (0.67–1.7). The dynamics of RAM tissue metabolite concentrations during the study period are shown in Figure 2.

The blood and RAM tissue glucose were stable throughout the observation period (Figure 2). Tissue pyruvate increased slightly during the first 6 hours; however, this increase did not reach a level of significance. Tissue lactate was high at the beginning of the study and decreased significantly during the observation period (*P* ≤ 0.008). Blood lactate was also elevated at the beginning of the study and normalized earlier than tissue lactate (*P* ≤ 0.02). The L/P ratio decreased significantly (*P* ≤ 0.0002) throughout the observation period and normalized 24 hours after enrollment in the study. The L/G ratio reached its maximum level at the 10th hour and subsequently started to decrease. Changes that occurred during the initial 36 hours were significant (*P* ≤ 0.006); however, the changes were not significant during the entire period of 72 hours.

Correlation analyses revealed an association between higher MAP and APP levels and lower tissue pyruvate concentrations and between the noradrenaline dose and RAM tissue pyruvate concentrations (Table 2). The elevated L/G ratio significantly correlated with a higher dose of noradrenaline (Table 2).

### 3.2. Glutamate and Glycerol

The baseline concentrations of tissue glutamate and glycerol were as follows: glutamate 38 (22–100) *μ*M and glycerol 251 (173–408) *μ*M. Figure 2 presents the dynamics of those metabolites.

RAM tissue glutamate, which was significantly increased at the beginning of the study, started to decrease after six hours and normalized by the end of the study (*P* ≤ 0.0001). A correlation analysis indicated an association between elevated IAP levels and higher tissue glutamate concentrations (Table 2).

RAM tissue glycerol concentrations did not change significantly during the study period; however, a simultaneous correlation analysis suggested that higher MAP and APP values associated with lower glycerol concentrations (Table 2).

## 4. Discussion

The present study investigated the changes of extracellular metabolites, glucose, pyruvate, lactate, glycerol, and glutamate, in the abdominal wall muscle tissue of critically ill patients with moderately increased intra-abdominal pressure. Our primary finding was that elevated IAP after initial resuscitation from shock was associated with anaerobic metabolism in the RAM tissue. Higher APP was associated with low pyruvate and glycerol concentrations, and elevated IAP was associated with higher tissue glutamate. These observations may indicate tissue ischemia and damage, despite modestly increased IAP.

The effects of IAH/ACS on end organs have been widely described; however, the pathophysiology is not well understood [13–15]. For example, although a clear rationale exists for APP, a recent consensus statement made no recommendation regarding its use in the resuscitation or management of critically ill patients [6]. Our finding of a negative correlation between APP, pyruvate, and glycerol indicates a likely relevance of APP as a resuscitation endpoint. The use of vasopressors contributed to keeping the MAP of our patients virtually unchanged and well above the target range of 75 mmHg. The fact that APP changed significantly and was associated with unfavorable changes supports the notion that IAP and APP should be considered when setting the targets for MAP and vasopressor therapy.

The present results corroborate, to some extent, with previous animal experiments. Meier et al. showed that an IAP over 20 mmHg in rats resulted in ischemic metabolic changes in RAM tissue detected by microdialysis and that these changes were evident before ACS was clinically apparent [16]. More recently, Benninger et al. conducted a microdialysis study in mechanically ventilated pigs with IAH/ACS and concluded that IAH (IAP 20 mmHg and 30 mmHg) induced ischemic metabolic changes that were first detected using microdialysis of the RAM compared to other intra-abdominal organs [8]. In the present clinical study, we did not observe so marked changes as demonstrated in animal studies. The species difference is certainly one possible explanation. Furthermore, it could be speculated that IAP of 20 mmHg or even 30 mmHg is a more serious insult in small animals (lower systemic blood pressure) than in humans (especially when considering respective APPs); therefore, such differences are to be expected. The clinical parallel studies by Meier et al. and Benninger et al. showed differences in ACS rather than IAH of grade I or II, with a corresponding IAP of 12 mmHg to 20 mmHg. Overt ACS, however, rarely occurs in clinical practice [3–5]. In those cases, deteriorations of cardiac, respiratory, and renal performance are usually clearly evident and determine the immediate need for life-saving treatment. The management of IAP below 20 mmHg is more controversial [3, 17] because there is no clear trigger when and to what extent to initiate treatment options of IAH [6].

In laparoscopic surgical patients, we observed that an IAP of 12-13 mmHg led to significant elevations of the L/P ratio during the procedure [7]. The present study results confirmed this finding: the L/P ratio and tissue lactate and glutamate are significantly elevated during IAH. When we compared the present results to the absolute values of RAM metabolites that are available from clinical studies, our patients appeared to have a markedly elevated L/P ratio and lactate and glycerol levels already at beginning of the study (Table 3). Hörer and coauthors measured intraperitoneal lactate, pyruvate, and glycerol in patients with an endovascular repair of a ruptured aortic aneurysm [19]. They observed an elevated L/P ratio and glycerol level (and IAP) in patients who were subjected to decompressive laparotomy because of clinically evident ACS.

The elevated tissue L/P ratio describes the cellular metabolic reaction to altered oxygen and glucose supplies [20]. The L/P ratio is a stronger marker of cell ischemia than lactate alone [21] because lactate may also be produced under aerobic conditions [22], whereas the L/P ratio is a specific marker of anaerobic conditions. A high L/P ratio at the beginning of our study strongly indicates hypoperfusion and anaerobic metabolism in RAM tissue during IAH.

Glucose is the basic energy substrate for the cells. Setälä and Gudaviciene found that tissue glucose and lactate concentrations, especially their relation L/G ratio, may indicate the presence of ischemia in a microvascular flaps. A high L/G ratio was associated with the amount of flap necrosis [23]. Our results are consistent with this finding that the L/G ratio reached its maximum level at the 10th hour, followed by stepwise decrease. Surprisingly, the significance of the L/G ratio trend appeared during the first 36 hours (*P* ≤ 0.006); however, the L/G ratio trend did not continue over the entire 72 hours of observation time (*P* ≤ 0.08).

Glutamate is a proteinogenic amino acid that may act as a neurotransmitter or metabolic substrate. Elevated extracellular concentrations may reflect excitotoxicity and membrane breakdown [24]. Glutamate release may be considered to be a surrogate of on-going ischemia [25, 26]. Microdialysis data are more reflective of local production than systemically circulating compounds [24]; therefore, the gradual decrease of glutamate may be interpreted as partial recovery from IAH during the study period.

Glycerol, which is most likely released from damaged cells because of ischemia and tissue injury, is related to the degradation of the glycerophospholipids of cell membranes [26]. In the present study, elevated pyruvate, lactate, and glutamate levels indicated RAM tissue hypoperfusion. We speculate that ischemia was not severe enough to cause cellular breakdown with concomitant glycerol release; therefore, we did not observe any changes in glycerol.

At the beginning of study, 7 of 10 patients were in shock. From our microdialysis results, it is unclear whether the observed changes occurred because of shock resolution or because of dynamics in IAP and APP. Control samples of nonabdominal origin, for example, from the extremities, would have been desirable to address this question; however, due to ethical and cost concerns, we inserted only one MD catheter per patient. This factor is the primary limitation of our study. Indirect support to our findings is derived from animal experiments by Meier and Benninger, who demonstrated that other tissues/locations are not influenced by IAP as fast as RAM is. Furthermore, the positive correlation between glutamate and IAP indicates the importance of IAP in observed metabolite changes.

## 5. Conclusions

The present study demonstrates that moderate IAH leads to RAM tissue anaerobic metabolism suggestive for hypoperfusion in critically ill patients. The correlation analysis supports the concept of using APP as a primary endpoint of resuscitation in addition to MAP and IAP. Feasibility of RAM microdialysis as a diagnostic tool for early-stage tissue damage requires additional investigation.

## Figures and Tables

**Table 1:** Patients' clinical characteristics at the baseline (at enrolment to study) and during the study period. Data are presented as median (interquartile range). *P ≤ 0.05 compared to baseline.

	Baseline	12–24 hours	24–48 hours	48–72 hours
IAP, (mmHg)	14.5 (12.5–17.8)	12.9 (10.4–14.3)*	11 (9.3–13.9)*	9.8 (9.3–14.9)*
MAP, (mmHg)	83 (75–89)	91 (82–95)	89 (80–96)	89 (87–919)
APP, (mmHg)	70 (58–74)	76 (66–85)*	72 (70–819)	81 (77–87)*
Heart rate, (beats/min)	106 (94–121)	107 (93–116)	92 (89–106)	90 (86–97)*
CVP, (mmHg)	19 (16–21)	17 (12–22)	15 (12–18)	14 (12–19)
Arterial glucose, (mmol/L)	7.6 (5.4–9.7)	8.0 (6.0–8.7)*	7.5 (6.6–9.4)	7.1 (6.2–8.9)
Arterial lactate, (mmol/L)	1.9 (1.3–3.75)	1.9 (1.1–2.9)	1.9 (0.8–2.2)	1.5 (0.8–1.8)
Arterial pH	7.42 (7.34–7.47)	7.44 (7.39–7.48)	7.44 (7.38–7.47)	7.43 (7.40–7.46)
Arterial pCO2, (mmHg)	38.3 (32.2–46.6)	40.6 (37.3–45.59)	41.4 (38.4–48.0)	42.3 (40.8–45.8)
SOFA score	7 (6–9)	6 (5–9)	8 (4–10)	6 (3–7)
Noradrenaline dose, (*μ*g/kg/min)	0.22 (0.11–0.35)	0.32 (0.11–0.53)	0.12 (0.07–0.27)	0.09 (0.03–0.20)*
CVI	3.5 (0–4.3)	4 (0–4.5)	2 (0–4.5)	3 (0–3.5)

IAP: intra-abdominal pressure; MAP: mean arterial pressure; APP: abdominal perfusion pressure; CVP: central venous pressure; SOFA: Sequential Organ Failure Assessment score; CVI: cumulative vasopressor index (a method for quantifying total amount of vasopressor support at a given time point) [18].

**Table 2:** Correlation analysis between RAM microdialysate metabolites and mean arterial pressure (MAP), intra-abdominal pressure (IAP), abdominal perfusion pressure (APP), noradrenaline dose (*μ*g/kg/min), and serum lactate concentration. Correlation is considered significant at *P* ≤ 0.05.

	MAP		IAP		APP		Noradrenaline	
	*R*	*P*	*R*	*P*	*R*	*P*	*R*	*P*
Glucose	−0.04	0.5	−0.1	0.3	0.02	0.9	0.1	0.1
**Pyruvate**	**−0.3**	**0.0003**	−0.08	0.4	**−0.3**	**0.009**	**0.2**	**0.04**
lactate	−0.1	0.09	−0.09	0.4	−0.4	0.3	0.1	0.08
**L/P ratio**	0.03	0.7	0.1	0.3	0.1	0.4	**0.2**	**0.02**
**L/G ratio**	0.09	0.2	−0.08	0.4	0.05	0.6	**0.6**	**0.0000**
**Glycerol**	**−0.2**	**0.008**	−0.04	0.7	**−0.2**	**0.03**	0.008	0.9
**Glutamate**	0.04	0.6	**0.2**	**0.02**	−0.02	0.8	−0.06	0.5

*R*: correlation coefficient.

**Table 3:** Parameters from different studies. Microdialysis catheter was either in RAM tissue (both Maddison et al. studies [7]) or in peritoneal cavity (Hörer et al. [19]).

Parameter	Patient group	Baseline/at inclusion	During/max values	At the end of study
IAP (mmHg)	Elective surgery	Normal^(1)^	12-13^(1)^	
	IAH, decompressed	16 (CI 12–20)^(2)^	19 (CI 12–23)^(2)^	
	IAH, nondecompressed	15 (CI 11–18)^(2)^	14 (CI 7–15)^(2)^	
	**ICU**	14.5 (12.5–17.8)*		9.8 (9.3–14.9)*

L/P ratio	Elective surgery	10.3 (7.1–15.5)^(1)^	20.2 (13.1–45.5)^(1)^	
	IAH, decompressed	20 (CI 17–25)^(2)^	24 (CI 17–36)^(2)^	
	IAH, nondecompressed	12 (CI 11–16)^(2)^	13 (CI 10–19)^(2)^	
	**ICU**	49 (36–54)*		24 (22–40)*

Glucose (mM)	Elective surgery	3.3 (0.1–4.4)^(1)^	0.9 (0.1–4.4)^(1)^	
	IAH, decompressed	11.0 (8.0–14.1)^(2)^	10.0 (6.4–13.6)^(2)^	
	IAH, nondecompressed	8.7 (6.1–11.3)^(2)^	8.9 (CI 6.0–12.5)^(2)^	
	**ICU**	4.1 (3.2–6.5)*		4.4 (3.0–6.3)*

Lactate (mM)	Elective surgery	1.1 (0.3–1.7)^(1)#^	1.7 (1.2–2.9)^(1)#^	
	IAH, decompressed	10.1 (4.8–12.4)^(2)^	6.5 (3.7–11.2)^(2)^	
	IAH, nondecompressed	2.7 (1.7–3.4)^(2)^	2.6 (CI 1.6–4.2)^(2)^	
	**ICU**	4.7 (2.7–10)*		3.4 (2.3–4.6)*

Pyruvate (*μ*M)	Elective surgery	85 (40–125)^(1)#^	112 (61–175)^(1)#^	
	**ICU**	129 (57–189)*		134 (75–167)*

Glycerol (*μ*M)	Elective surgery	103 (65–169)^(1)^	326 (144–730)^(1)^	
	IAH, decompressed	274.6 (172.5–475.4)^(2)^	245.5 (117.8–512.9)^(2)^	
	IAH, non-decompressed	121.7 (62.4–212.7)^(2)^	135 (CI 76.2–272.9)^(2)^	
	**ICU**	251 (173–408)*		330 (174–361)*

^(1)^Maddison et al. 2012 [7], 6 patients RAM microdialysis (MD) during elective laparoscopic surgery; ^#^part of the data unpublished.

^(2)^Hörer et al. 2013 [19], 15 patients Intraperitoneal MD during endovascular repair of RAA, non-decompressed (9) and decompressed (6) patients.

*Current study, 10 ICU patients with IAH.
